# Draft genome and description of *Merdibacter massiliensis* gen.nov., sp. nov., a new bacterium genus isolated from the human ileum

**DOI:** 10.1038/s41598-019-44343-8

**Published:** 2019-05-28

**Authors:** Hussein Anani, Rita Abou Abdallah, Nisrine Chelkha, Anthony Fontanini, Davide Ricaboni, Morgane Mailhe, Didier Raoult, Pierre-Edouard Fournier

**Affiliations:** 1 Aix Marseille Univ, Institut de Recherche pour le Développement (IRD), Service de Santé des Armées, AP-HM, UMR Vecteurs Infections Tropicales et Méditerranéennes (VITROME), Institut Hospitalo-Universitaire Méditerranée Infection, Marseille, France; 2 Aix-Marseille Université, Institut de Recherche pour le Développement (IRD), UMR Microbes Evolution Phylogeny and Infections (MEPHI), Institut Hospitalo-Universitaire Méditerranée-Infection, Marseille, France; 3 Special Infectious Agents Unit, King Fahd Medical Research Center, King Abdulaziz University, Jeddah, Saudi Arabia

**Keywords:** Bacteriology, Clinical microbiology

## Abstract

We used phenotypic, genomic and phylogenetic information following the taxono-genomics approach to demonstrate that strain Marseille–P3254, isolated from an ileal sample of a 76-year old woman who underwent upper and lower digestive tract endoscopy for esophagitis and colonic polyp, is representative of a novel bacterial genus within the family *Erysipelotrichaceae* in the phylum *Firmicutes*. It is an anaerobic Gram-negative bacterium without catalase and oxidase activities. The genome of strain Marseille–P3254 is 2,468,496-bp long with a 40.1% G + C content. This new bacterium is most closely related to *Eubacterium dolichum*, with which it shares 90.7% 16S rRNA sequence similarity. In addition, genomic comparison using the digital DNA–DNA hybridization and OrthoANI analyses between the novel organism and the *E. dolichum* type strain revealed identities of 25.2 and 68.91%, respectively. The major fatty acids were C_16: 0_, C_18: 1n9_ and C_18: 0_. Based on these data, we propose the creation of the new genus *Merdibacter* gen. nov., with strain Marseille-P3254^T^ (=CSUR P3254 = DSM 103534) being the type strain of the new species *Merdibacter massiliensis* gen. nov., sp. nov.

## Introduction

A thorough knowledge of the gut microbiota composition appears essential to understand many aspects of health and diseases in humans. Culturomics, a new approach to study and decipher the human microbiota, based on the diversification of culture conditions and complementary to 16S rRNA metagenomics, has enabled the culture of more than 500 new bacterial species to date^1–3^. Most of these new species were characterized using the taxono-genomics strategy that combines phenotypic characteristics and whole genome sequencing analysis^4^.

**Figure 1:**
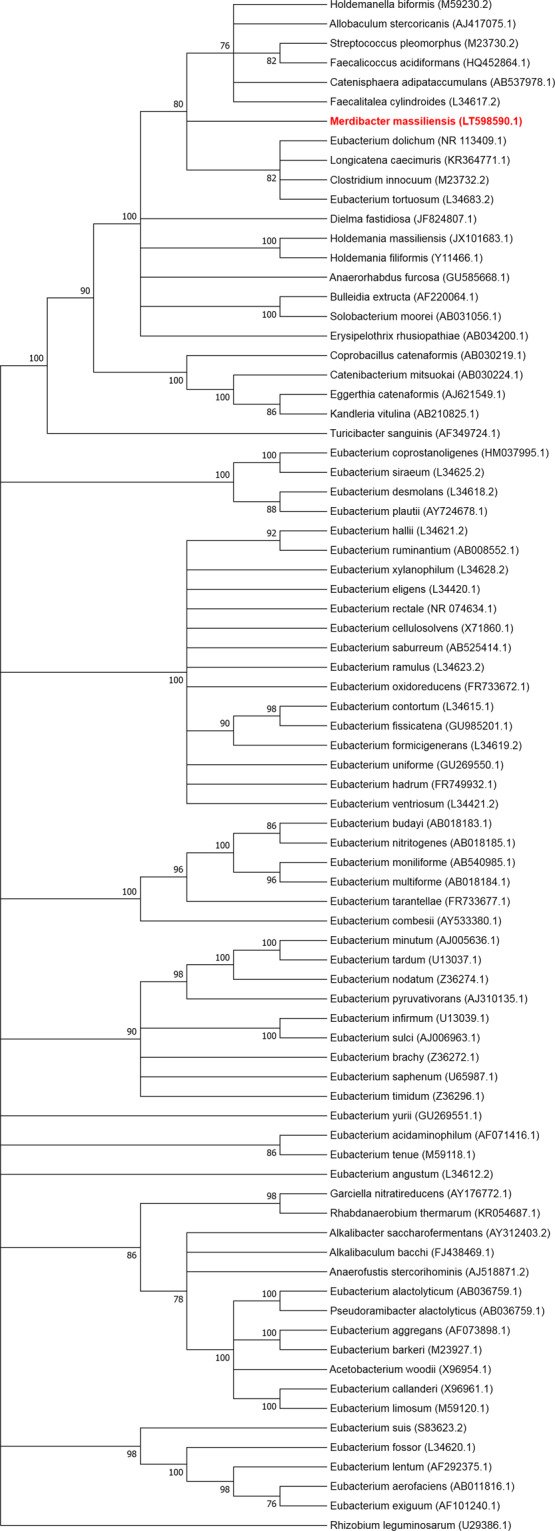
16S rRNA-based phylogenetic tree highlighting the position of *Merdibacter massiliensis* gen. nov. sp., nov., strain Marseille-P3254 (red) relative to other closely related bacterial taxa within the family *Erysipelotrichaceae* as well as members of the family *Eubacteriaceae*. Genbank accession numbers are presented in parentheses. Sequences were aligned using Muscle v3.8.31 with default parameters and phylogenetic relationship inferred using the Maximum Likelihood method, with 1,000 bootstrap replicates, within the MEGA software version 7.0. Only values above 70% were indicated. *Rhizobium leguminosarum* was used as outgroup.

In 2016, we isolated the new bacterial strain Marseille-P3254 (=CSUR P3254 = DSM 103534), from an ileal sample of a 76-year-old patient who underwent upper and lower digestive tract endoscopy^5^ for esophagitis and colonic polyp. Matrix-assisted desorption ionization–time of flight mass spectrometry (MALDI-TOF MS)^6^ failed to identify this isolate at the species level. The strain, predicted to be affiliated with members of the family *Erysipelotrichaceae*, is most closely related to *Eubacterium dolichum* with which it exhibits a 16S rRNA sequence similarity of 90.7% (Fig. 1). The family *Erysipelotrichaceae* is composed of 13 genera with validly published names (*Allobaculum, Bulleidia, Catenibacterium, Catenisphaera, Coprobacillus Eggerthia, Erysipelothrix, Faecalicoccus, Faecalitalea, Holdemanella, Holdemania, Solobacterium* and *Turicibacter*)^7^. *Eubacterium dolichum*^8^ was initially described as a member of the family *Eubacteriaceae*^9^. However, a recent study has questioned this classification and proposed that this species be classified within a new genus in the family *Erysipelotrichaceae*^10^. The aim of the current study was to describe and determine the exact taxonomic position of strain Marseille-P3254, for which the name *Merdibacter massiliensis* gen. nov., sp. nov. is proposed, on the basis of a polyphasic characterization of its phenotypic properties and genome comparison with closely related bacterial taxa.

## Results

### Strain identification and classification

Strain Marseille-P3254 was isolated from the ileal content of a 76-year-old woman who underwent upper and lower digestive tract endoscopy for esophagitis and colonic polyp. The patient gave an informed and written consent and the study was approved by the ethics committee of the Institut Hospitalo-Universitaire Mediterranee Infection under number 2016-010. As no microorganism grew from the negative control, we are confident that *Merdibacter massiliensis* was not an experiment contamination. Strain Marseille-P3254 could not be identified by our systematic MALDI-TOF MS screening as the score was 1.763, suggesting that the corresponding species was not in the database (Figure S1). Moreover, strain Marseille-P3254 exhibited a 90.70% 16S rRNA sequence similarity with *Eubacterium dolichum* strain JCM 10413^T^ (GenBank accession no. NR_113409), the phylogenetically closest bacterium with standing in nomenclature (Fig. 1). As this value is lower than the 95% threshold defined by Stackebrandt and Ebers for delineating a new genus, strain Marseille-P3254 was considered as representative of a putatively new genus within the family *Erysipelotrichaceae* in the phylum *Firmicutes*.

### Phenotypic characteristics

**Figure 2:**
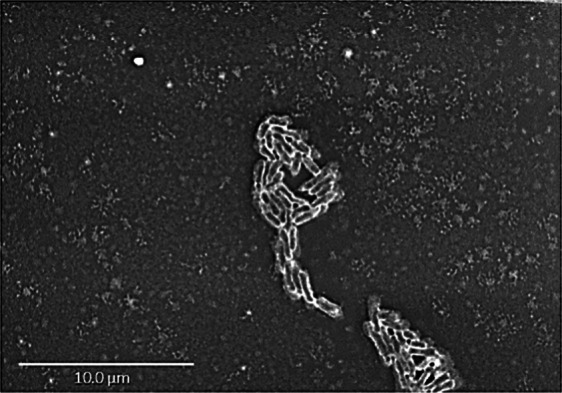
Scanning electron microscopy of *Merdibacter massiliensis* gen. nov., sp. nov., strain Marseille-P3254 using a Tabletop microscope TM 4000 plus (Hitachi, Tokyo, Japan). The scale bar represents 10 µm.

Growth was observed on 5% sheep blood-enriched Columbia agar (bioMérieux) at 37 °C and 45 °C after 5 days of incubation. Colonies from strain Marseille-P3254 showed neither pigmentation nor haemolysis. They were circular with a diameter of 0.5 to 1.5 mm, and transparent. Bacterial cells were Gram-negative, non-motile rods with a length of 1.50 to 2.78 µm and a width of 0.3 to 0.5 µm, as determined by electronic scanning microscopy (Fig. 2). Strain Marseille-P3254 grew only in anaerobic conditions. The sporulation test (20 minutes at 80 °C) was negative. In addition, this bacterium had no oxidase and catalase activities.

Using an API ZYM strip, a positive reaction was observed for alkaline and acid phosphatases but negative reactions were observed with esterase, esterase lipase, lipase, leucine arylamidase, valine arylamidase, cystine arylamidase, trypsin, α-chymotrypsin, naphtol-AS-BI-phosphohydrolase, α-galactosidase, ß-galactosidase, ß-glucuronidase, α-glucosidase, ß-glucosidase, N-acetyl-ß-glucosaminidase, α-mannosidase and α-fucosidase. Using an API 20NE strip, negative reactions were obtained for reduction of potassium nitrate, indole production from tryptophan, glucose fermentation, arginine hydrolysis, urea, aesculin, gelatin, p-nitrophenyl-ßD-galactopyranoside, and assimilation of glucose, arabinose, mannose, mannitol, N-acetyl-glucosamine, maltose, gluconate, caprate, adipate, malate, citrate and phenyl-acetate. Using an API 50 CH strip, strain Marseille-P3254 was able to metabolize glycerol, D-galactose, D-glucose, D-fructose, D-mannose, methyl-αD-glucopyranoside, N-acethylglucosamine, D-maltose, D-lactose, D-saccharose, D-trehalose, D-turanose, D-tagatose and potassium 5-Ketogluconate. However, negative reactions were obtained with erythritol, D-arabinose, L-arabinose, D-ribose, D-xylose, L-xylose, D-adonitol, methyl-ßD-xylopyranoside, L-sorbose, L-rhamnose, dulcitol, inositol, D-mannitol, D-sorbitol, methyl-αD-mannopyranoside, amygdalin, arbutin, esculin, salicin, D-cellobiose, D-melibiose, inulin, D-melezitose, D-raffinose, starch, glycogen, xylitol, gentiobiose, D-lyxose, D-fucose, L-fucose, D-arabitol, L-arabitol, potassium gluconate and potassium 2-ketogluconate.

According to the French Microbiology Society, susceptibility tests showed that strain Marseille-P3254 was susceptible to cefoxitin, linezolid, vancomycin, trimethoprim-sulfamethoxazole, metronidazole and ciprofloxacin, but resistant to ticarcillin-clavulanic acid, cefepime, ceftriaxone, erythromycin, fosfomycin, rifampin, amikacin and teicoplanin.

By comparison with closely related taxa, strain Marseille-P3254 differed in a combination of negative catalase activity and positive galactose metabolism (Table 1). The major fatty acids were Hexadecanoic acid (34%) and 9-Octadecenoic acid (34%). Significant abundances of C16:0, C18:1n9 and C18:0 were also described. In addition, lighter aliphatic chains were detected such as C10:0, C12:0, C14:0 and C15:0 (Table S1).

**Table 1:** Compared phenotypic characteristics of studied species.

Characteristics	MM	ED	FC	CI	DF	HB	HM	SP
Gram stain	−	+	+	+	−	+	+	+
Production of Catalase	−	−	−	−	−	−	+	−
Oxidase	−	−	−	Na	−	−	−	−
Nitrate reductase	−	−	−	−	−	−	−	−
Gelatin hydrolysis	−	−	−	+	na	+	na	−
Utilisation of L-Arabinose	−	−	−	na	−	+	−	−
D-Galactose	+	−	−	na	−	−	+	−
D-Fructose	+	+	−	na	−	−	+	−
D-Mannose	+	−	+	+	−	+	+	+
D-Rhamnose	−	−	−	na	−	−	−	−
D-Mannitol	−	−	−	+	−	+	+	−
D-Sorbitol	−	−	−	−	na	−	+	−
Amygdalin	−	−	−	na	na	−	+	−
Arbutin	−	na	na	na	na	−	+	−
Salicin	−	−	−	na	na	+	+	−
D-Cellobiose	−	−	−	+	−	−	+	−
D-Maltose	+	+	−	+	−	−	+	−
D-Lactose	+	−	−	−	−	−	+	−
D-Trehalose	+	+	−	+	−	−	−	−
Inulin	−	na	na	na	na	−	na	−
Starch	−	−	−	na	na	−	na	−
Glycogen	−	−	−	na	na	−	na	−
Xylitol	−	na	na	na	−	−	−	−
D-Arabitol	−	na	na	na	−	−	−	−
D-Glucose	−	−	+	+	−	+	+	+
Raffinose	−	−	+	+	−	+	−	−
D-Xylose	−	+	−	+	−	+	−	−

ED: *Eubacterium dolichum;* FC: *Faecalitalea cylindroides;* DF: *Dielma fastidiosa;* HB: *Holdemanella biformis;* HM: *Holdemania massiliensis:* SP: *Streptococcus pleomorphus;* MM: *Merdibacter massiliensis;* CI: *Clostridium innocuum*. Data for reference strains^5,8,10,39–41^. +: positive reaction, −: negative reaction, na: data not available.

### Genome sequencing information and genome properties

**Figure 3:**
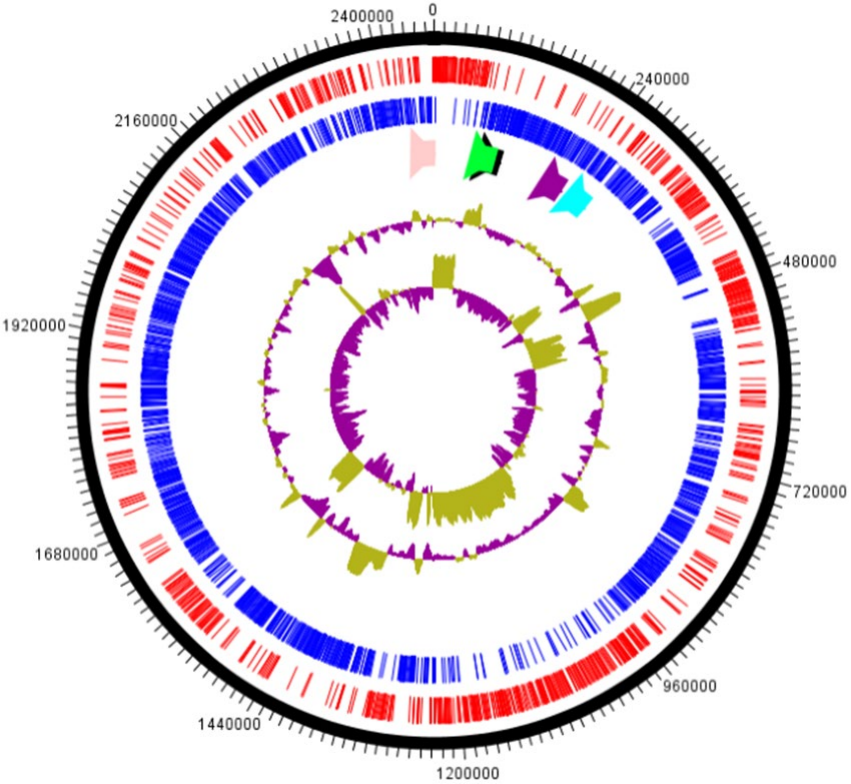
Graphical circular map of the genome from strain Marseille-P3254. From outside to the center: genes on the forward strand colored in dark blue, genes on the reverse strand colored in red, the five resistance genes identified by comparison with the ARG-ANNOT database colored in pink, green, black, purple and light blue, G + C content and G + C skew.

The genome size of strain Marseille-P3254 was 2,468,496-bp long with a 40.1% G + C content. It was assembled into 24 contigs. Of the 2,375 predicted genes, 2,315 were protein-coding genes and 60 were RNAs (one complete rRNA operon, three additional 5S rRNAs, 50 tRNAs and 4 non-coding RNAs). A total of 1,643 genes (69.1%) were assigned a putative function and 672 genes (28.2%) were annotated as hypothetical proteins. The genome properties and distribution of genes into COGs functional categories are detailed in Table S2. The *in silico* resistome of this multidrug resistant strain includes genes coding resistance to tetracycline (*tetW*), aminoglycoside (*ant6*), macrolide-lincosamide-streptogramin B (*lsa*) and oxazolidinone (*oxzln*) (Fig. 3).

In the genome from *Merdibacter massiliensis*, we identified genes coding enzymes involved in the pentose phosphate pathways (13 genes), including *aldoa* (fructosebiphosphate aldolase, 1 copy), *dera* (deoxyribose-phosphate aldolase, 1 copy), *rpel* (ribulose phosphate 3-epimerase, 2 copies), *pfkl* (6-phosphofructokinase, 1 copy), *pgd* (6-phosphogluconate dehydrogenase, 1 copy), *pgm1* (phosphoglucomutase, 2 copies), *pgm2* (phosphopentomutase, 1 copy), *rpib* (ribose-5-phosphate isomerase B, 1 copy), *taldo1* (transaldolase, 1 copy) and *tkt* (transketolase, 2 copies). We also identified sugar phosphotransferase systems (52 predicted genes), including were *ulaB* (Ascorbate-specific PTS system, 5 copies), *bglF* (PTS system beta-glucoside-specific, 7 copies), *frwD* (PTS system fructose-like, 3 copies), *ptsg* (PTS system glucose-specific, 6 copies), *malX* (PTS system maltose-specific, 3 copies), *manX* (PTS system mannose-specific, 15 copies), *gmuB* (PTS system oligo-beta-mannoside-specific, 1 copy), *sorA* (PTS system sorbose-specific, 10 copies), *treP* (PTS system trehalose-specific, 1 copy) and *dhaM* (PTS-dependent dihydroxyacetone kinase, 1 copy). In addition, we identified several predicted proteins known to play a role in vitamin metabolism, including folate family ECF transporter S component, tetrahydrofolate ligase, 5-formyltetrahydrofolate cyclo-ligase, methylenetetrahydrofolate (folates), biotin transporter BioY, acetyl-CoA carboxylase biotin carboxylase subunit, acetyl-CoA carboxylase biotin carboxyl carrier protein, biotin–[acetyl-CoA-carboxylase] ligase (biotin), bifunctional riboflavin kinase/FAD synthetase (riboflavin) and cobalamin biosynthesis protein CobW (cobalamin).

*In silico* search for virulence factors showed the presence of six proteins conferring a potential pathogenicity with high identity percentage. One of these proteins exhibited 97% identity (100% sequence coverage) with its ortholog (Transcription regulator Immr) in the pathogenic bacterium *Clostridium difficile* strain R2029. In addition, a predicted protein exhibited 100% similarity with the virulence-associated protein E from *Streptococcus suis*. The other four predicted proteins were conjugal transfer protein Tcpe (86% identity), transcription regulator yobd (85%), antirestiction protein ArdA (84%) and excisionate HTH (81%).

**Figure 4:**
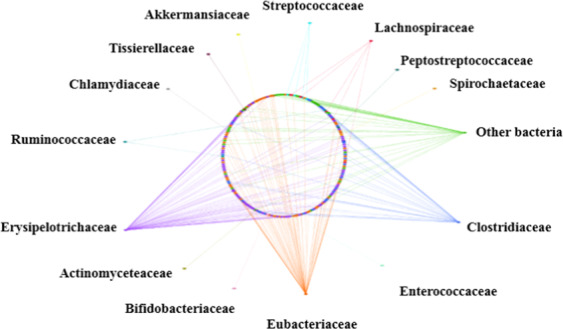
Network showing the origin of predicted protein-coding genes in strain Marseille-P3254 according to bacterial families. A total of 714 protein-coding genes were likely to be acquired vertically or from closely related species within the family *Erysipelotrichaceae*, and 1601 genes were likely to be gained by lateral gene transfer from members of other bacterial families.

A total of 714 protein-coding genes were likely to be acquired vertically or from closely related species, as their closest orthologs belonged to members of the family *Erysipelotrichaceae* to which strain Marseille-P3254 also belonged (Fig. 4). In addition, 1601 genes were likely to be gained by lateral gene transfer (LGT) from members of other bacterial families (Fig. 4). Most lateral gene transfer (LGT)-acquired genes were obtained from *Erysipelotrichaceae* (30.84%) followed by other bacteria (17.14%), *Clostridiaceae* (17.10%) and *Eubacteriaceae* (15.42%).

### Comparison with closely related bacterial strains

**Figure 5:**
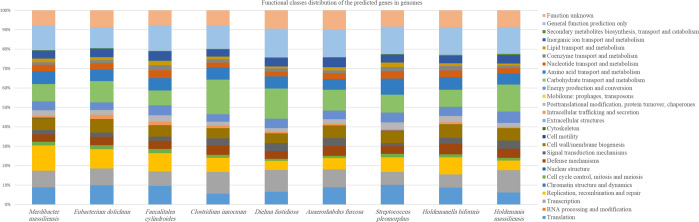
Distribution of functional classes of predicted genes according to the clusters of orthologous groups of proteins of *Merdibacter massiliensis* gen. nov., sp. nov., strain Marseille-P3254 and other compared bacterial taxa.

**Figure 6:**
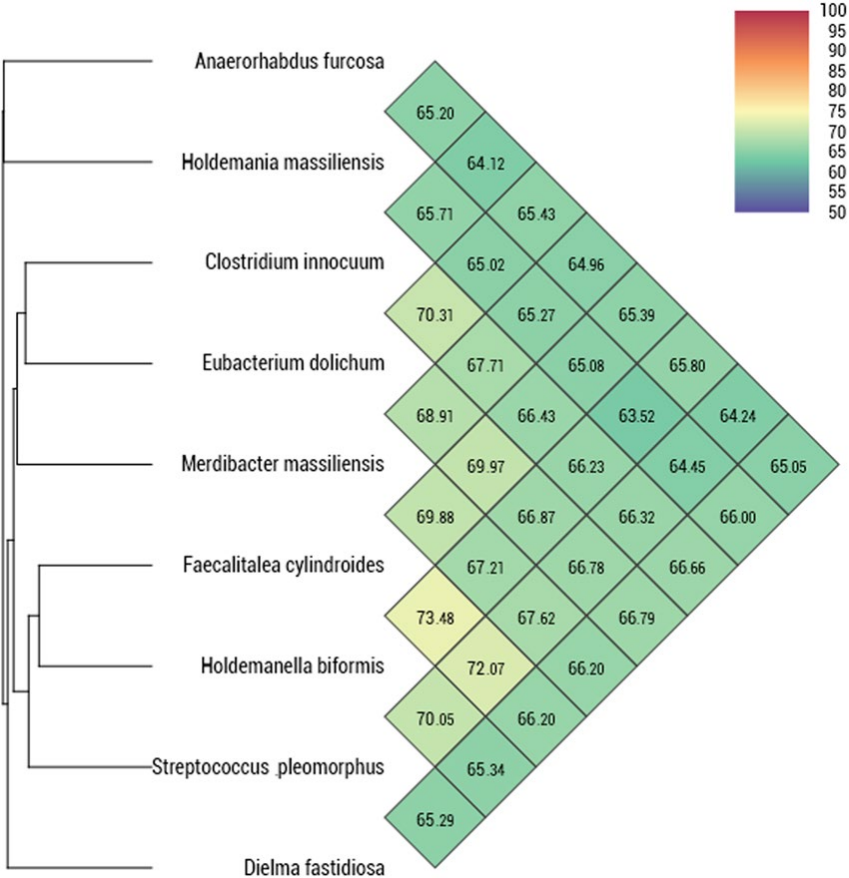
Heatmap generated with OrthoANI values calculated using the OAT software between *Merdibacter massiliensis* gen. nov., sp. nov., strain Marseille-P3254 and other closely related taxa with standing in nomenclature.

The genome of strain Marseille P3254 was compared to the available genomes of eight closely related bacterial type strains (Table S3). Core-genome-based phylogenetic relationships of strain Marseille-P3254 and the closest species with standing in nomenclature are presented in Fig. S2. The distribution of genes into COG categories was similar in all nine compared genomes (Fig. 5). Strain Marseille-P3254 shared 975, 584, 966, 835, 905, 860, 1,137 and 754 orthologous genes with *Eubacterium dolichum*, *Faecalitalea cylindroides*, *Dielma fastidiosa*, *Holdemanella biformis*, *Streptococcus pleomorphus*, *Holdemania massiliensis*, *Clostridium innocuum* and *Anaerorhabdus furcosa*, respectively (Table 2). Moreover, MAGi (Marseille Average Genomic identity)^11^ analysis showed that AGIOS (Average Genomic Identity of Orthologous gene Sequences)^4^ values ranged from 49.2% between *Anaerorhabdus furcosa* and *Holdemania massiliensis*, to 52.39% between *Anaerorhabdus furcosa* and *Holdemanella biformis*, among studied genera with standing in nomenclature. Regarding strain Marseille-P3254, the range of AGIOS value varied from 50.25% with *Holdemania massiliensis* to 52.14% with *Dielma fastidiosa* (Table 2). Strain Marseille-P3254 was closer to *Dielma fastidiosa*, with 52.14% genomic identity, but shared more orthologous genes (1,137) with *Clostridium innocuum*. Finally, dDDH (digital DNA-DNA hybridization)^12,13^ estimation of strain Marseille-P3254 against the available genomes for type strains of species, with standing in nomenclature ranged from 18.7% between *Dielma fastidiosa* and *Streptococcus pleomorphus*, to 41.1% between *Eubacterium dolichum* and *Faecalitalea cylindroides* (Table S4). These values are lower than 70% the cutoff used for delineating prokaryotic species, thus confirming that this strain represents a genus distinct from these other bacterial strains. Ortho Average nucleotide identity^14^ (OrthoANI) values ranged between 63.52 and 73.48% (orthoANI value lower than 80.5%) confirming again that this strain is distinct from these other tested bacterial taxa (Fig. 6). Cumulatively, the above data suggest that strain Marseille-P3254 represents a novel species within a new genus in the family *Erysipelotrichaceae*, for which the name *Merdibacter massiliensis* gen. nov., sp. nov., is proposed.

**Table 2:** Numbers of orthologous proteins shared between genomes (upper right) and AGIOS values (lower left). The numbers of proteins per genome are indicated in bold.

	ED	FC	DF	HB	MM	SP	HM	CI	AF
ED	**2,190**	507	955	769	975	811	839	1,098	744
FC	51.55%	**1,971**	490	574	584	631	459	612	424
DF	51.31%	51.065%	**3,497**	779	966	820	1,083	1,209	916
HB	51.61%	51.735%	51.20%	**2,392**	835	895	727	934	663
MM	51.77%	51.20%	52.14%	51.47%	**2,411**	905	860	1,137	754
SP	51.64%	51.34%	51.13%	51.38%	51.29%	**2,005**	742	990	722
HM	50.12%	49.87%	50.60%	49.61%	50.25%	50.49%	**3,486**	1,097	917
CI	50.71%	50.48%	50.79%	50.46%	50.81%	50.65%	50.64%	**4,702**	932
AF	51.65%	51.57%	51.07%	52.39%	51.42%	51.47%	49.20%	50.21%	**2,404**

ED: *Eubacterium dolichum;* FC: *Faecalitalea cylindroides;* DF: *Dielma fastidiosa;* HB: *Holdemanella biformis;* HM: *Holdemania massiliensis:* SP: *Streptococcus pleomorphus;* MM: *Merdibacter massiliensis;* CI: *Clostridium innocuum;* AF: *Anaerorhabdus furcosa*.

## Discussion

Based on the diversification of culture conditions, the new microbial approach “culturomics” aiming at deciphering the complexe diversity of the human microbiota, allowed isolation of more than 1,500 different human bacterial species over the past 6 years, including more than 500 new species^1,15^. Using the taxono-genomics concept combining the genomic and phenotypic properties of a putative new taxa^15^, we have characterized a new bacterial species representing a new genus within the family *Erysipelotrichaceae* in a human ileal specimen. One of the human gut microbiota characteristics is the richness of the enzymes related to central metabolism (like the pentose phosphate pathway) and sugar phosphotransferase systems (PTS)^16,17^. In addition, the metabolic phenotype observed for strain Marseille-P3254 was similar to that of *Eubacterium dolichum*, its closest phylogenetic neighbor and also a gut microorganism. As a consequence, as *Merdibacter massiliensis* exhibits metabolic characteristics that enable harvesting nutrients and energy from the diet, and are consistent with a role in metabolic homeostasis in the human gut, we believe that it may survive in the human gut^18–20^. Thus, we propose the creation of the new genus and species *Merdibacter massiliensis* gen. nov., sp. nov., a member of the *Erysipelotrichaceae* family, closely related to *E. dolichum* that was recently proposed to belong to a new genus within this family^10^. Bioinformatic analysis predicted that our strain was a putative pathogen. This hypothesis was supported by the presence of several proteins associated to pathogenesis (hemolysin III, type II toxin-antitoxin system Hica), all of which were acquired from to the family *Clostridiaceae* (Fig. 4). In addition, strain Marseille-P3254 shared several protein-coding genes with *Streptococcus suis*^21^, a pathogen causing gastro-intestinal tract infections in human, including a virulence-associated protein E. However, the ileal lavage being part of the exploration of a colonic polyp, no clear association of *M*. *massiliensis* with pathogenesis can be inferred in this patient. The network of *M. massiliensis* exhibited a remarkable inheritance of genes from bacterial families distinct from its parent family (Fig. 4). The Digital Protologue TaxoNumber (http://imedea.uibcsic.es/dprotologue/index.php) of *M. massiliensis* gen. nov., sp. nov. is TA00782.

### Description of *Merdibacter* gen. nov

*Merdibacter* (Mer.di.ba’c.ter, L. masc. n. *Merdibacter*, composed of merdi from *merda*, ‘excrement’ and *bacter*, ‘a rod’. *Merdibacter*, ‘a rod from faeces’). Cells are anaerobic, Gram-negative, nonmotile and asporogenous rods. Catalase and oxidase activities are negative.

### Description of *Merdibacter massiliensis* gen. nov., sp. nov

*Merdibacter massiliensis* (mas.si.li.en’sis. L. masc. adj. massiliensis, of Massilia, the Latin name of Marseille where strain Marseille-P3254 was first isolated).

In addition, the description features of the genus, cells have a length of 1.5 µm to 2.78 µm and a width of 0.3 to 0.5 µm. Colonies grown on 5% sheep blood-enriched Columbia agar (bioMérieux) are circular and transparent after 5 days of incubation in anaerobic atmosphere, varying in size from 0.5 to 1.5 mm in diameter. Growth occurs at 37 °C (optimum) and 45 °C. Cells grow anaerobically only. Using an API ZYM strip, a positive reaction is observed for alkaline and acid phosphatases but negative reactions are observed with esterase, esterase lipase, lipase, leucine arylamidase, valine arylamidase, cystine arylamidase, trypsin, α-chymotrypsin, naphtol-AS-BI-phosphohydrolase, α-galactosidase, ß-galactosidase, ß-glucuronidase, α-glucosidase, ß-glucosidase, N-acetyl-ß-glucosaminidase, α-mannosidase and α-fucosidase. Using an API 20NE strip, negative reactions are obtained for reduction of potassium nitrate, indole production from tryptophan, glucose fermentation, arginine hydrolysis, urea, aesculin, gelatin, p-nitrophenyl-ßD-galactopyranoside, and assimilation of glucose, arabinose, mannose, mannitol, N-acetyl-glucosamine, maltose, gluconate, caprate, adipate, malate, citrate and phenyl-acetate. Using an API 50 CH strip, strain Marseille-P3254 was able to metabolize glycerol, D-galactose, D-glucose, D-fructose, D-mannose, methyl-αD-glucopyranoside, N-acethylglucosamine, D-maltose, D-lactose, D-saccharose, D-trehalose, D-turanose, D-tagatose and potassium 5-Ketogluconate. However, negative reactions are obtained with erythritol, D-arabinose, L-arabinose, D-ribose, D-xylose, L-xylose, D-adonitol, methyl-ßD-xylopyranoside, L-sorbose, L-rhamnose, dulcitol, inositol, D-mannitol, D-sorbitol, methyl-αD-mannopyranoside, amygdalin, arbutin, esculin, salicin, D-cellobiose, D-melibiose, inulin, D-melezitose, D-raffinose, starch, glycogen, xylitol, gentiobiose, D-lyxose, D-fucose, L-fucose, D-arabitol, L-arabitol, potassium gluconate and potassium 2-ketogluconate. The most abundant fatty acids are hexadecanoic acid (C_16:0_), 9-Octadecenoic acid (C_18:1n9_) and Octadecanoic acid (C_18: 0_). The genome is 2,468,496-bp long and its G + C content is 40.1%.

The type strain, Marseille-P3254^T^, isolated from the ileum of a patient, was deposited in the CSUR and DSMZ collections under accession numbers CSUR P3254 and DSM 103534, respectively. The 16S rRNA and genome sequences are available in GenBank under accession numbers LT598590 and FTLC00000000, respectively.

## Materials and Methods

### Strain isolation and phenotypic tests

As per our culturomics procedure^2^, the fresh ileal wash sample was collected in sterile vial and then inoculated in an anaerobic blood culture vial (Becton Dickinson, Pont de Claix, France) enriched with 5 mL of sterile sheep blood (BioMérieux) and 5 mL of filter-sterilized (Thermo Fisher Scientific, Vilbon-sur-Yvette, France) rumen fluid (3 successive filtrations using filters with 0.8 µm, 0.45 µm, and 0.2 µm pore sizes). In parallel, an anaerobic blood culture vial (Becton Dickinson) enriched with 5 mL of sterile sheep blood (bioMérieux) was inoculated with 5 mL of filter-sterilized (Thermo Fisher Scientific) rumen fluid as a negative control to verify the sterility of the nutrient. After 7 days of incubation at 37 °C, the suspension was inoculated on 5% sheep blood-enriched Columbia agar (BioMérieux, Marcy l’Etoile, France) in anaerobic atmosphere (anaeroGEN, Oxoid, Dardilly, France). Isolated colonies were identified using MALDI-TOF mass spectrometry (MS) protein analysis and a Microflex spectrometer (Bruker Daltonics, Bremen, Germany)^22^. Spectra from strain Marseille-P3254 were imported into the MALDI BioTyper software (version 2.0, Bruker) and analyzed by standard pattern matching (with default parameter settings) against 11805 bacterial spectra in the Bruker database (7854 spectra) enriched with spectra from bacteria isolated in our laboratory as part of the culturomics study (3951 spectra). Interpretation of the scores was performed as previously described^23^.

Moreover, the 16S rRNA gene was sequenced using the fD1-rP2 primer pair as previously described^24^, using a 3130-XL sequencer (Applied Biosciences, Saint Aubin, France). A phylogenetic tree was obtained using the Maximum Likelihood method and Kimura 2-parameter within the MEGA 7 software^25^. Different growth temperatures (20, 28, 37, 45 and 55 °C) were tested. Growth of strain Marseille-P3254 was tested under different atmospheres (anaerobic, aerobic and microaerophilic) (CampyGEN, Oxoid). API ZYM, API NE and API 50CH strips (BioMérieux) were used to evaluate the biochemical properties of the strain according to the manufacturer’s instructions. For scanning electronic microscopy, a colony was collected from agar and immersed into a 2.5% glutaraldehyde fixative solution. The slide was gently washed in water; air dried and examined with approximately 60 centimeters in height and 33 cm in width to evaluate bacterial structure on a TM4000 microscope. The standard disc method was applied for antimicrobial susceptibility testing according to the French Microbiology Society. Finally, cellular fatty acid methyl ester (FAME) analysis was performed by GC/MS. Two samples were prepared with approximately 25 mg of bacterial biomass per tube harvested from several culture plates. Briefly, fatty acid methyl esters were separated using an Elite 5-MS column and monitored by mass spectrometry (Clarus 500 - SQ 8S, Perkin Elmer, Courtaboeuf, France)^26^. GC/MS analyses were carried out as previously described^27,28^. Spectral database search was performed using MS Search 2.0 operated with the Standard Reference Database 1A (NIST, Gaithersburg, USA) and the FAME mass spectral database (Wiley, Chichester, UK).

### DNA extraction and genome sequencing

Genomic DNA (gDNA) of strain Marseille-P3254 was extracted in two steps: a mechanical treatment was first performed by acid-washed glass beads (G4649-500g Sigma) using a FastPrep BIO 101 instrument (Qbiogene, Strasbourg, France) at maximum speed (6.5 m/sec) for 90 sec. Then, after a 2.5 hour lysozyme incubation at 37 °C, DNA was extracted using an EZ1 biorobot (Qiagen) with EZ1 DNA Tissue kit. The elution volume was 50 µL. gDNA was quantified by a Qubit assay with the high sensitivity kit (Life technologies, Carlsbad, CA, USA) at 313 ng/µl.

Genomic DNA was sequenced on a MiSeq sequencer (Illumina Inc, San Diego, CA, USA) with the Mate Pair strategy. The gDNA was barcoded in order to be mixed with 11 other projects with the Nextera Mate Pair sample prep kit (Illumina).

The Mate Pair library was prepared with 1.5 µg of genomic DNA using the Nextera Mate Pair Illumina guide. The gDNA sample was simultaneously fragmented and tagged with a Mate Pair junction adapter. The profile of the fragmentation was validated on an Agilent 2100 BioAnalyzer (Agilent Technologies Inc, Santa Clara, CA, USA) with a DNA 7500 labchip. DNA fragments ranged in size from 1 kb up to 11 kb with an optimal size at 2.38 kb. No size selection was performed and 114.4 ng of tagmented fragments were circularized. The circularized DNA was mechanically sheared to small fragments with an optimal at 993 bp on the Covaris device S2 in microtubes (Covaris, Woburn, MA, USA). The library profile was visualized on a High Sensitivity Bioanalyzer LabChip (Agilent Technologies Inc, Santa Clara, CA, USA) and the final concentration library was measured at 10.51 nmol/l.

The library was normalized at 2 nM and, after a denaturation step and dilution at 15 pM, loaded onto the reagent cartridge and then onto the instrument along with the flow cell. Automated cluster generation and sequencing run were performed in a single 39 hour run in a 2 × 151 bp format.

Total information of 2.8 Gbp was obtained with 274 K/mm^2^ cluster density with a cluster passing quality control filters of 97.6% (5,537,000 passing filter paired reads). Within this run, the index representation for strain Marseille-P3254 was determined to 9.48%. The 524,686 paired reads were assembled using the SPAdes version 3.10.1 software^29^. The option “careful” was used in order to reduce the number of mismatches and short indels. Default parameters were applied for K values, *i.e*., k-mer values of 127, 99, 77, 55, 33, and 21. SSPACE^30^ and GapFiller^31^ were used to combine contigs with default parameters. Finally, manual finishing was performed by using similarity searches based on BLAST searches, and synteny blocks were detected by progressive alignment using the Mauve software^32^.

### Genome annotation and genome comparison

The genome was annotated as previously described^23^. In addition, we compared the genome from strain Marseille-P3254 to those of the closely related species *Eubacterium dolichum* strain JCM 10413^T^ (accession number ABAW00000000), *Faecalitalea cylindroides* strain JCM 10261^T^ (AWVI00000000), *Dielma fastidiosa* strain DSM 26099^T^ (CAEN00000000), *Holdemanella biformis* strain DSM 3989^T^ (ABYT00000000), *Streptococcus pleomorphus* strain DSM 20574^T^ (ATUT00000000), *Holdemania massiliensis* strain DSM 26143^T^ (CALK00000000), *Clostridium innocuum* strain DSM 1286^T^ (AGYV00000000) and *Anaerorhabdus furcosa* strain ATCC 25662^T^ (FUWY00000000). For this, we used the Genome-to Genome Distance Calculator (GGDC) web server available at http://ggdc.dsmz.de to estimate the overall similarity among compared genomes and to replace the wet-lab DNA–DNA hybridization (DDH) by a digital DDH (dDDH)^12,13^. The average nucleotide identity at the genomic level was also estimated using the orthoANI^14^ and AGIOS^4,33^ software. Antibiotic resistance genes (ARG) were searched using the ARG-ANNOT database and Bio-Edit interface^34^. Assembled sequences were searched against the ARG-ANNOT database under moderately stringent conditions (e-value of 10^−5^) for the *in silico* ARG prediction. These putative ARGs were further confirmed through a BLAST search against non-redundant (nr) database in GenBank.

The presence of pathogenesis-related proteins was investigated using PathogenFinder 1.1^35^. Predicted protein sequences of strain Marseille-P3254 were used as queries to search the NCBI GenBank non-redundant protein sequence database. These results were formatted to generate a network of protein sequences using the Cytoscape tool^36^. Finally, predicted protein sequences were searched against KEGG PATHWAY^37^ and PATRIC 3.5.31^38^ databases for the screening of genes involved in metabolic pathways.

## Supplementary information

Supplementary data
