# Supplementary material for: Draft genome and description of Merdibacter massiliensis gen.nov., sp. nov., a new bacterium genus isolated from the human ileum
Source: Sci Rep. 2019 May 28;9:7931. doi: 10.1038/s41598-019-44343-8 (PMC6538751; doi:10.1038/s41598-019-44343-8)
Supplement: Supplementary file 1 — Supplementary data [file 41598_2019_44343_MOESM1_ESM.docx]

**Title: Draft genome and description of *Merdibacter massiliensis* gen.nov., sp. nov., a new bacterium genus isolated from the human ileum**

**Authors :** Hussein ANANI^1^, Rita ABOU ABDALLAH^1^, Nisrine CHELKHA^2^, Anthony FONTANINI^2^, Davide RICABONI^2^, Morgane MAILHE^2^, Didier RAOULT^2, 3^, Pierre-Edouard FOURNIER^1^*

**Supplementary data**

**Figure S1:** Reference MALDI-TOF MS spectrum from strain Marseille-P3254.


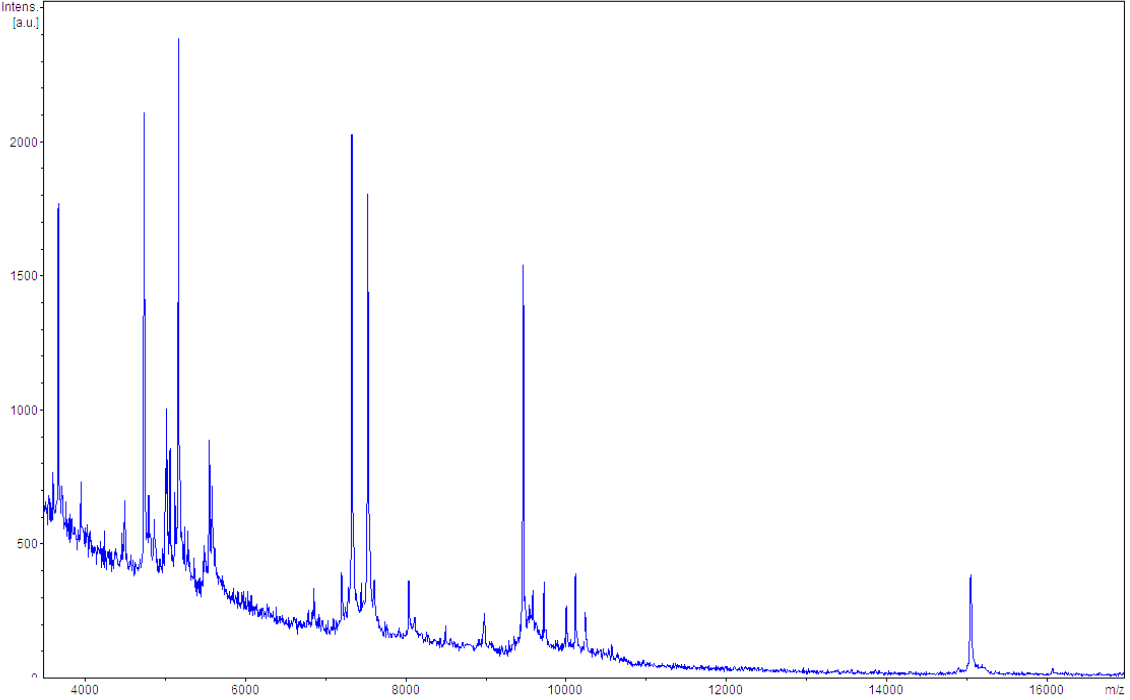


**Figure S2:** Phylogenetic tree based on 171 core genes highlighting the position of *Merdibacter massiliensis* (red) relative to other closely related bacterial taxa. Genbank genome accession numbers are presented in parentheses. The annotated GFF3 file of reference genomes was used as matrix in Roary version 3.10.2 on galaxy online site (<http://www.usegalaxy.org.au>) choosing a minimum percentage blastp identity of 50%. Core-genome alignement was converted from FASTA to PHYLIP format which has uploaded in ATGC Montpellier bioinformatics platform (<http://www.atgc-montpellier.fr/phyml>). Using the MEGA version 7.0 software, core genome sequences were realigned using Muscle v3.8.31 with default parameters and phylogenetic relationships inferred using the Maximum Likelihood method with 1,000 bootstrap replicates.

**
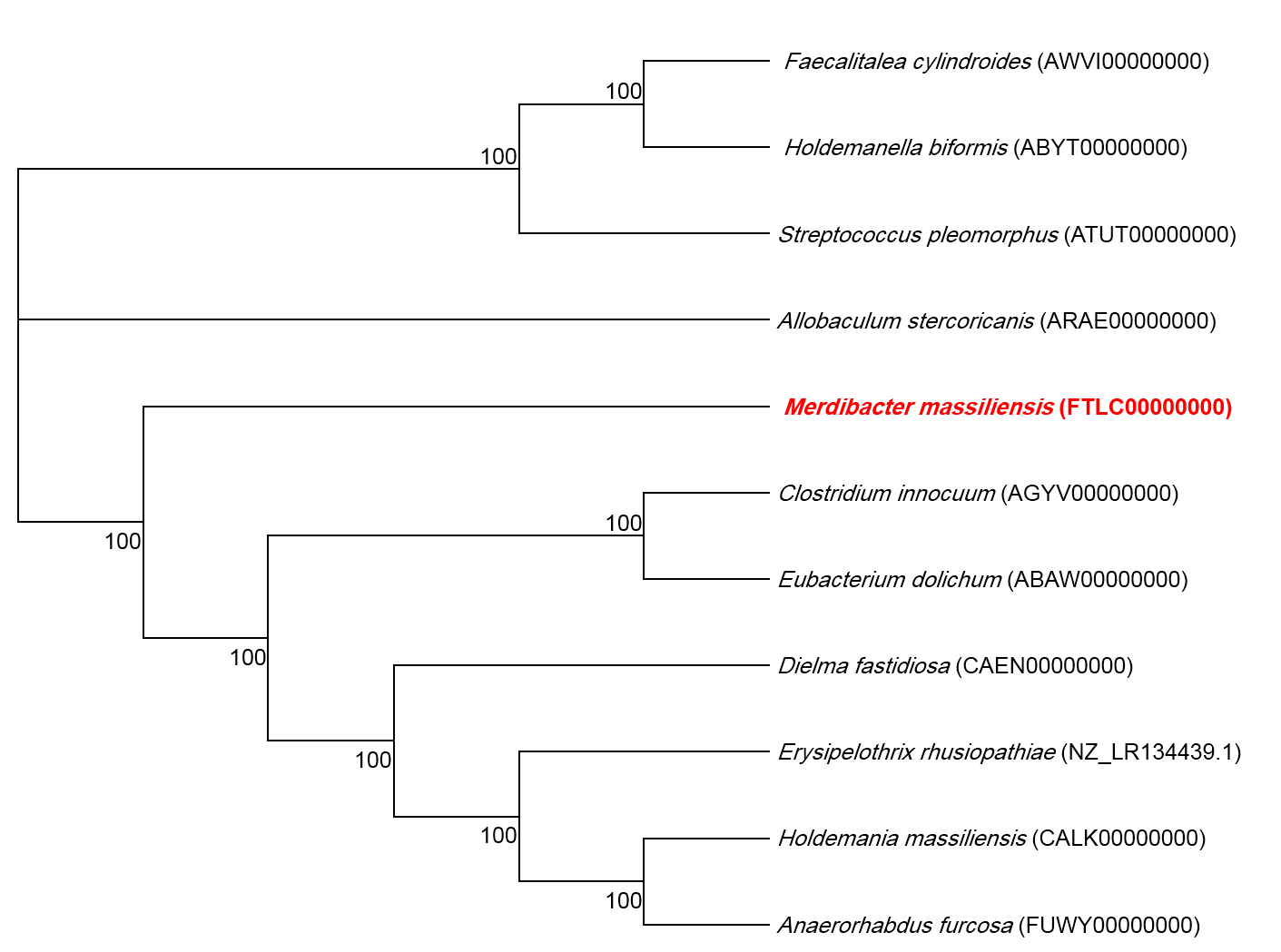
**

**Table S1:** Cellular fatty acid composition (%) of strain Marseille-P3254.

| **Fatty acids** | **Name** | **Mean relative % (a)** |
| --- | --- | --- |
| 16:0 | Hexadecanoic acid | 33.7±0.7 |
| 18:1n9 | 9-Octadecenoic acid | 33.5±0.8 |
| 18:0 | Octadecanoic acid | 15.3±0.2 |
| 18:2n6 | 9,12-Octadecenoic acid | 6.1±0.3 |
| 18:1n7 | 11-Octadecenoic acid | 4.5±0.1 |
| 14:0 | Tetradecanoic acid | 3.4±0.1 |
| 16:1n7 | 9-Hexadecenoic acid | 1.2±0.2 |
| 17:0 | Heptadecanoic acid | TR |
| 17:1n8 | 9-Heptadecenoic acid | TR |
| 15:0 | Pentodecanoic acid | TR |
| 10:0 | Decanoic acid | TR |
| 17:0 anteiso | 14-methyl-Hexadecanoic acid | TR |
| 17:0 iso | 15-methyl-Hexadecanoic acid | TR |
| 12:0 | Dodecanoic acid | TR |
| 15:0 anteiso | 12-methyl-tetradecanoic acid | TR |

^a^Mean peak area percentage ; TR = trace amounts < 1%

**Table S2:** Numbers of genes of strain Marseille-P3254 associated with the 25 general COG functional categories

| Code | Value | % value | Description |
| --- | --- | --- | --- |
| J | 145 | 8.86 | Translation |
| A | 0 | 0 | RNA processing and modification |
| K | 141 | 8.61 | Transcription |
| L | 213 | 13.01 | Replication, recombination and repair |
| B | 0 | 0 | Chromatin structure and dynamics |
| D | 30 | 1.83 | Cell cycle control, mitosis and meiosis |
| Y | 0 | 0 | Nuclear structure |
| V | 63 | 3.85 | Defense mechanisms |
| T | 35 | 2.14 | Signal transduction mechanisms |
| M | 101 | 6.17 | Cell wall/membrane biogenesis |
| N | 3 | 0.18 | Cell motility |
| Z | 0 | 0 | Cytoskeleton |
| W | 0 | 0 | Extracellular structures |
| U | 18 | 1.1 | Intracellular trafficking and secretion |
| O | 45 | 2.75 | Posttranslational modification, protein turnover, chaperones |
| X | 0 | 0 | Mobilome: prophages, transposons |
| C | 76 | 4.64 | Energy production and conversion |
| G | 146 | 8.92 | Carbohydrate transport and metabolism |
| E | 108 | 6.6 | Amino acid transport and metabolism |
| F | 54 | 3.3 | Nucleotide transport and metabolism |
| H | 22 | 1.34 | Coenzyme transport and metabolism |
| I | 30 | 1.83 | Lipid transport and metabolism |
| P | 66 | 4.03 | Inorganic ion transport and metabolism |
| Q | 5 | 0.31 | Secondary metabolites biosynthesis, transport and catabolism |
| R | 206 | 12.58 | General function prediction only |
| S | 130 | 7.94 | Function unknown |
| - | 678 | 41.42 | Not in COGs |

**Table S3:** Genomic characteristics of *M. massiliensis* gen. nov., sp. nov*.* and the 8 most closely related bacterial taxa for which genome sequences are available.

| Type strains | Accession number | Size (Mb) | GC % | Gene content |
| --- | --- | --- | --- | --- |
| *Merdibacter massiliensis* | FTLC00000000 | 2.46 | 40.1 | 2,375 |
| *Eubacterium dolichum* | ABAW00000000 | 2.19 | 38.1 | 2,215 |
| *Faecalitalea cylindroides* | AWVI00000000 | 1.95 | 34.7 | 2,005 |
| *Clostridium innocuum* | AGYV00000000 | 4.77 | 43.4 | 4,737 |
| *Dielma fastidiosa* | CAEN00000000 | 3.57 | 40.0 | 3,466 |
| *Streptococcus pleomorphus* | ATUT00000000 | 1.99 | 39.0 | 2,046 |
| *Holdemanella biformis* | ABYT00000000 | 2.41 | 33.8 | 2,433 |
| *Holdemania massiliensis* | CALK00000000 | 3.75 | 47.0 | 3,533 |
| *Anaerorhabdus furcosa* | FUWY00000000 | 2.40 | 32.1 | 2,423 |

**Table S4:** dDDH values obtained by comparison of all studied genomes.

|  | MM | ED | FC | HB | CI | DF | SP | HM | AF |
| --- | --- | --- | --- | --- | --- | --- | --- | --- | --- |
| MM  100 | | 25.20±4.8 | 30.60±4.9 | 19.40±4.6 | 23.80±4.8 | 20.10±4.6 | 22.60±4.7 | 22.10±4.7 | 28±4.8 |
| ED  100 | | | 41.10±5.1 | 30.10±4.9 | 23.10±4.8 | 22.20±4.7 | 20.20±4.6 | 21.40±4.7 | 26.30±4.9 |
| FC  100 | | | | 26.40±4.8 | 24.80±4.8 | 21.30±4.7 | 24.80±4.8 | 25.50±4.8 | 20.00±4.6 |
| HB  100 | | | | | 28.10±4.9 | 21.20±4.7 | 18.90±4.5 | 25.60±4.9 | 19.80±4.6 |
| CI  100 | | | | | | 22.00±4.7 | 22.70±4.7 | 26.30±4.8 | 29.30±4.8 |
| DF  100 | | | | | | | 18.70±4.6 | 36.40±5 | 20.60±4.7 |
| SP  100 | | | | | | | | 26.10±4.8 | 23.20±4.8 |
| HM  100 | | | | | | | | | 28.80±4.8 |
| AF  100 | | | | | | | | | |

dDDH: digital DNA-DNA hybridization ; MM: *Merdibacter massiliensis;* ED: *Eubacterium dolichum;* FC: *Faecalitalea cylindroides;* HB: *Holdemanella biformis;* CI: *Clostridium innocuum ;* DF: *Dielma fastidiosa;* SP: *Streptococcus pleomorphus;* HM: *Holdemania massiliensis;* AF: *Anaerorhabdus furcosa.*
